# When to Initiate Weight Loss Medications in the NAFLD Population

**DOI:** 10.3390/diseases6040091

**Published:** 2018-09-30

**Authors:** Eric R. Yoo, Sandy Sallam, Brandon J. Perumpail, Umair Iqbal, Neha D. Shah, Waiyee Kwong, George Cholankeril, Donghee Kim, Aijaz Ahmed

**Affiliations:** 1 Department of Medicine, Santa Clara Valley Medical Center, San Jose, CA 95128, USA; ericryoo@gmail.com; 2 Division of Gastroenterology and Hepatology, Stanford University School of Medicine, Stanford, CA 94305, USA; ssallam@stanfordhealthcare.org (S.S.); NeShah@stanfordhealthcare.org (N.D.S.); WKwong@stanfordhealthcare.org (W.K.); georgetc@stanford.edu (G.C.); dhkimmd@stanford.edu (D.K.); 3 Drexel University College of Medicine, Philadelphia, PA 19129, USA; brandonperumpail@gmail.com; 4 Department of Medicine, Mary Imogene Bassett Hospital, Cooperstown, NY 13326, USA; umairiqbal_dmc@hotmail.com

**Keywords:** NAFLD, NAFL, NASH, weight loss medication, weight loss, nutrition

## Abstract

Nonalcoholic fatty liver disease (NAFLD) is characterized by histological evidence of hepatic steatosis, lobular inflammation, ballooning degeneration and hepatic fibrosis in the absence of significant alcohol use and other known causes of chronic liver diseases. NAFLD is subdivided into nonalcoholic fatty liver (NAFL) and nonalcoholic steatohepatitis (NASH). NAFL is generally benign but can progress to NASH, which carries a higher risk of adverse outcomes including cirrhosis, end-stage liver disease, hepatocellular carcinoma and death if liver transplantation is not pursued in a timely fashion. Currently, lifestyle modifications including healthy diet and increased physical activity/exercise culminating in weight loss of 5% to >10% is the cornerstone of treatment intervention for patients with NAFLD. Patients with NAFLD who fail to obtain this goal despite the help of dietitians and regimented exercise programs are left in a purgatory state and remain at risk of developing NASH-related advance fibrosis. For such patients with NAFLD who are overweight and obese, healthcare providers should consider a trial of FDA-approved anti-obesity medications as adjunct therapy to provide further preventative and therapeutic options as an effort to reduce the risk of NAFLD-related disease progression.

## 1. Defining NAFLD: NAFL and NASH

Nonalcoholic fatty liver disease (NAFLD) is defined by evidence of hepatic steatosis by imaging or histology in the absence of other causes of chronic liver disease or long-term exposure to a steatogenic medication. NAFLD is subdivided into 1) nonalcoholic fatty liver (NAFL) characterized by hepatic steatosis without histological evidence of inflammation or fibrosis; and 2) nonalcoholic steatohepatitis (NASH) which demonstrates varying degrees of hepatic steatosis, lobular inflammation, ballooning degeneration and hepatic fibrosis In majority of patients, NAFLD is associated with metabolic comorbidities such as diabetes mellitus, obesity, and hyperlipidemia; not surprisingly, the most common cause of death in patients with NAFLD is cardiovascular disease [1]. The management of NAFLD thus targets not only the liver disease, but also its associated metabolic comorbidities. Patients with NAFLD have increased overall mortality compared to matched-control populations without the disease [2,3]. There exists a perception that NAFLD in obese individuals is worse than NAFLD in individuals who are lean, but a recent study suggests that metabolic dysfunction in patients with biopsy-proven NAFLD were similar in both obese and non-obese (and non-diabetic) populations [4]. This group is coined as having “Lean NAFLD,” which describes a condition of impaired insulin resistance, increased cardiovascular risk, and increased hepatic lipid storage in individuals who are not obese [4]. This subcategory of NAFLD should be in consideration when managing patients who may not fit the stereotypical descriptions often associated with patients with NAFLD and metabolic syndrome.

## 2. Prevalence of NAFLD and NASH

Not all patients with NAFLD have NASH and not all patients with NAFL develop NASH. The estimated prevalence of NASH in NAFLD patients who had a liver biopsy without specific clinical indications is 6.67% (95% CI, 2.17–18.73) to 29.85% (95% CI, 22.72–38.12) and that of NASH among NAFLD patients who had a liver biopsy for clinical indications is 59.1% (95% CI, 47.55–69.73). The prevalence of NASH in the general population is estimated to range between 1.5% and 6.45% [5].

## 3. Diagnosing NAFLD: NAFL and NASH

Currently, liver biopsy remains the current gold standard in diagnosing NAFLD and differentiating NAFL from NASH. This diagnostic method is, however, limited by cost, sampling error, its invasive nature, and procedure-related morbidity and mortality. Various imaging modalities can help detect NAFLD, but no method is superior over another when differentiating NAFL and NASH. Such imaging methods include ultrasound, computed tomography (CT), magnetic resonance imaging (MRI), magnetic resonance elastography (MRE), and magnetic resonance spectroscopy (MRS). The ultrasound is accessible and relatively cost-effective when compared to other imaging modalities listed. A meta-analysis showed that the sensitivity and specificity of ultrasound in diagnosing NAFLD were 85 and 94 percent, respectively, when compared to liver biopsies; unfortunately, sensitivity decreases when assessing morbidly obese patients [6,7,8]. CT and MRI can identify steatosis, but they do not have adequate sensitivity to detect inflammation or fibrosis [9]. MRS has high diagnostic accuracy and is not significantly impacted by demographics or coexisting hepatic conditions [10]; however, it is not readily available. MRE has high accuracy for the diagnosis of significant or advanced fibrosis/cirrhosis independent of BMI and etiology of liver disease [11].

Noninvasive, non-imaging measures assessing steatohepatitis and fibrosis in NAFLD have been limited in utility thus far. Laboratory tests such as aminotransferases are often abnormal in NALFD but are insufficient for the diagnosis of NAFL or NASH. Noninvasive non-imaging markers with high sensitivity and specificity for NAFL and NASH have yet to be developed and remain of high interest by academics, clinicians, and the biotechnology industry alike. Two developing areas of interest that may impact how NAFLD is diagnosed and managed in the future are genetics and machine learning. Recent studies on genetic and epigenetic modifiers of NAFLD progression suggest that the I148M PNPLA3 variant is the major common genetic determinant of NAFLD [12]. Models utilizing such genetic-markers and machine learning may be utilized in the future to help clinicians predict which patients with NAFLD are at higher risk for liver fibrosis and progression to NASH [12]. 

The degree of hepatic steatosis and fibrosis is important in characterizing the degree of severity of NAFLD and in differentiating between NAFL and NASH. This is important because NAFL is generally benign while NASH carries a higher risk for adverse outcomes such as cirrhosis and end-stage liver disease. As a result, patients with NASH have an increased liver-related mortality rate. NASH is currently the second leading indication for liver transplantation (LT) in the United States (US) and is projected to become the number one cause of LT in the United States [13,14,15].

## 4. Management of NAFLD and NASH

The first-line management for treatment of metabolic syndrome and obesity is lifestyle modification. Lifestyle modification (diet, exercise, or both) has been shown to reduce the incidence of cardiovascular mortality, all-cause mortality, and diabetes, particularly in those with impaired glucose tolerance [16]. Generally, those with NAFLD are treated with lifestyle modification targeting metabolic syndrome and obesity, with the goal of losing ≥5% of body weight. A meta-analysis of eight randomized controlled trials showed that in adults who were able to lose ≥5% of body weight had improvement in hepatic steatosis and those who lost ≥7% body weight experienced improvement in the NAFLD Activity Score (NAS), a semi-quantitative assessment scoring system used to measure changes in liver histology of patients [17]. The current guideline recommends a hypocaloric diet (daily reduction by 500–1000 kcal) and moderate intensity exercise to sustain weight loss over time, and reiterates the importance of weight loss, highlighting that weight loss of greater than (7–10%) is needed to improve the majority of the histo-pathological features of NASH [1,18].

The benefits of weight loss in NAFLD may not be limited to changes in disease alone. A prospective cohort study suggested that patients with NAFLD can experience significant improvements in their quality of life by losing weight, and suggest that this correlation does not appear specific to biochemical improvements [19]. While it is easy to stress the importance of weight loss to individuals with NAFLD, it is a difficult goal to achieve and maintain for many. Success does not depend on effort alone. Factors such as the composition of diet with optimization of both micro- and macro-nutrients and increased frequency of encounters in the clinic are important in helping weight reduction for patients with NAFLD [20,21]. 

Diet is one critical component of NAFLD management and one model that has been studied extensively is the Mediterranean diet. This is a nutritional model that primarily consists of eating vegetables and fresh fruit, olive oil, unrefined cereals, and nuts. This diet also consists of eating fish, white meat, and legumes in moderation and limiting processed meats, red meats, and sugar. As the study of nutrition and health continue to expand, groups have developed models to better assess the effects of nutrition on liver diseases such as NAFLD [22].

Many studies have looked at the relationship between NAFLD progression and commonly consumed products such as green tea and coffee. Coffee increases an antioxidant enzyme periredoxin-1 which helps reduce reactive oxygen species and decrease oxidative stress on hepatocytes [23]. It also contains polyphenols that are similar to silymarin, which can increase the production of antioxidant proteins [23]. One prospective study observed a protective effect of coffee on fibrosis progression and an inverse correlation between increased coffee intake and clinically significant fibrosis [24]. A meta-analysis showed that coffee drinkers had a decreased risk of NAFLD and that regular daily consumption correlated with decreased risk of fibrosis [25,26]. Green tea, like coffee, has been shown to have beneficial effects in NAFLD. In green tea, epigallocatechin-3-gallate (EGCG) is thought to be the main therapeutic agent in NAFLD; EGCG decreases hepatic inflammation through reduction of cycloosxygenase-2, prostaglandin 2, NF-kB, and toll-like receptor 4 [27,28,29,30]. Polyphenols and EGCG in green tea also have antioxidative effects [31]. A 12-week study in which 80 patients were randomized to receive 500 mg of green tea extract or placebo once daily showed improvements measured in the treatment group, specifically inflammatory markers, insulin resistance, adiponectin, aminotransferases, and regression of fatty liver on ultrasound examinations [25,32].

## 5. NAFLD Purgatory

NAFL without progression can be benign. And many cases do not progress into NASH. However, time remaining in a state of NAFL is time remaining at risk of progressing into NASH. Patients with NAFL who are unable to sustain a hypocaloric diet with moderate-intensity exercise remain at risk of progressing into NASH. Patients with NAFL who fail to lose ≥5% of their body weight also remain at risk of progressing into NASH. Studies can help delineate what factors are correlated with increased weight reduction in patients with NAFLD, but many patients with NAFLD have comorbidities such as cardiovascular disease, rheumatologic disorders, and socioeconomic circumstances that limit the level of sustained and constant physical activities recommended by current guidelines. Many patients with NAFLD also fail to reach ≥5% of their body weight despite consultation with nutritionists and dietitians. Surgical intervention with bariatric surgery can be considered in patients with a BMI of 40 kg/m^2^ or greater or a BMI of 35 kg/m^2^ or greater and one obesity-related comorbidity [33]. 

Bariatric surgery was found to improve comorbid disease in most patients and improve long-term survival and cardiovascular disease related death. It was also shown to significantly improve prevalence and severity of steatosis and ballooning at 1 and 5 years following bariatric surgery [34]. Moreover, a meta-analysis by Bower et al. showed that majority of patients who underwent bariatric surgery appeared to show improvement in the histopathological features of steatosis, inflammation, and ballooning [35]. One prospective study conducted in France looked at the effect of bariatric surgery in patients with NASH and found that NASH had disappeared in 85% of patients one year after surgery [36]. Moreover, the authors found that patients who underwent laparoscopic gastric banding lost less weight and had a higher proportion of persistent NASH than those who underwent gastric bypass. Long-term effects of bariatric surgery in morbidly obese patients with NASH have yet to be established. Much of the literature has focused on the effects of bariatric surgery on NASH, but not on NAFL. Patients with NAFL who fail weight loss and are ineligible for bariatric surgery are left in a state of purgatory, risking progression into NASH and fibrosis. 

## 6. Pharmacological Intervention

Patients with NAFL who develop NASH are eligible for pharmacological intervention through clinical trials. Such novel options remain unavailable for patients with NAFL. Unsurprisingly, there have been many drugs developed for the purposes of weight reduction and targeting obesity. Weight loss pharmacological therapy is recommended for those with a BMI of ≥30 Kg/m^2^ or ≥27 kg/m^2^ with metabolic comorbidities such as diabetes or hypertension. The addition of pharmacotherapy has been shown to result in greater weight loss and weight loss maintenance compared with lifestyle modifications alone [37]. Currently there are 9 pharmacological interventions approved by the US Food and Drug Administration (FDA) (Table 1) that can be used for weight loss: Phentermine, diethylpropion, benzphetamine, phendimetrizine, orlistat, phentermine/topiramate extended release (ER), lorcaserin, bupropion/naltrexone, and liraglutide. To ensure efficacy of weight loss drugs, the FDA requires approval benchmarks for weight loss drug trials. Generally, the drug must demonstrate mean weight loss difference of at least 5% compared to placebo. Of note, there have been anti-obesity medications withdrawn from the market by the FDA (Table 2).

These medications are intended as adjuncts to continued efforts at lifestyle changes, including diet and exercise in adults. Prior to initiating obesity pharmacological interventions, a review of the patient’s medication history is recommended to assess for other medications that could be contributing to weight gain, and adjustments should be made when possible. Use of these medications in patients who are pregnant or planning to become pregnant is not recommended due to harmful or unknown fetal effects. 

Phentermine, diethylpropion, benzphetamine, and phendimetrazine are all sympathomimetic amines that decrease appetite through central nervous system (CNS) effects, including stimulation of the hypothalamus to release norepinephrine and dopamine. Of all four, phentermine is the most commonly prescribed. They are approved only as short-term treatments of up to 12 weeks. Dose adjustment is recommended in those with creatinine clearance ≤30 mL/min. They are contraindicated in patients with various comorbidities, including hypersensitivity to other sympathomimetic amines, history of cardiovascular disease (including arrhythmias, coronary artery disease, stroke, heart failure, and uncontrolled hypertension), history of drug abuse, and monoamine oxidase inhibitor (MAOI) use. Side effects include dry mouth, constipation, insomnia, tachycardia, and elevated blood pressure. Due to abuse potential, they are classified as schedule IV medications by the Food and Drug Administration (FDA). A 28-week randomized control trial compared phentermine, topiramate ER, and the combination of the two, and found that 46% of subjects assigned to phentermine lost ≥5% of initial body weight [38].

Phentermine/topiramate is a combination drug that is also FDA-approved and used for weight management. Both components suppress appetite, but topiramate has additional satiety enhancement through various CNS effects. The dosing is titrated up over a period of 12 weeks. Dose adjustment is recommended in those with creatinine clearance ≤50 mL/min and those with Child-Pugh class B. It is contraindicated in those with glaucoma, severe depression, recent stroke or CV event, nephrolithiasis (due to risk of topiramate-induced metabolic acidosis/kidney stones), and MAOI use. Due to teratogenicity effects, the FDA requires a Risk Evaluation and Mitigation Strategy (REMS) program that requires provider training and pharmacy certification for prescribing and dispensing of the drug. It is also a schedule IV drug due to its abuse potential. This combination therapy was compared with placebo and after one year, the percent weight loss was greater in the experimental group (8–10% of baseline body weight) versus 1.2% in the control group. A 52-week extension of the first trial showed significantly reduced body weight (9.3% vs. 10.5% vs. 1.8%) in low dose, high dose, and placebo groups, respectively [39]. If weight loss of 5% from baseline is not seen after 12 weeks at the highest dose, it should be discontinued through a taper (every other day for at least 1 week).

The combination bupropion-naltrexone was approved by the FDA in 2014. Naltrexone is a pure opioid agonist and bupropion is a weak inhibitor of the neuronal reuptake of norepinephrine and dopamine. The exact effects of this combination leading to weight loss is not fully understood, however, it is presumably due to the action on areas of the brain involved in the regulation of the hypothalamus (appetite regulation) and the mesolimbic dopamine pathway (reward). Some contraindications to bupropion-naltrexone include uncontrolled hypertension, chronic opioid, opiate agonist (methadone), or partial agonist (buprenorphine) use, acute opioid withdrawal, seizure disorder or conditions that lower the seizure threshold, use of MAOIs, pregnancy, and those receiving linezolid or IV methylene blue. Compared to placebo, the combination pill has been shown to reduce weight by an average of 5% greater than placebo at 1 year [40]. The dose is titrated up once weekly over a period of 4 weeks. Maximum dose cutoffs are recommended in those with moderate to severe renal impairment and hepatic impairment. After 16 weeks from initiation, if a 5% weight loss is not seen, continuation should be re-evaluated. If discontinued, it should be tapered off.

Loracaserin is a serotonin 5-HT2C receptor agonist and anorexiant approved by the FDA in 2012. Loracaserin is thought to activate the serotonin 5-HT2C receptors in the hypothalamus, resulting in increased satiety. Dosing is twice daily and does not involve titration. An extended release once daily formulation is available. It is contraindicated in individuals with severe depression, established cardiac valvular disease, those taking MAOIs, and creatinine clearance <30 mL/min. Use in patients with severe hepatic impairment (Child-Pugh class C) has not been studied. In a yearlong randomized study of 4008 patients, those taking lorcaserin lost 5% of body weight compared to placebo [41]. If 5% weight loss is not achieved within 12 weeks of initiation, efficacy is unlikely, and the medication should be discontinued.

Orlistat was approved by the FDA in 1999 for long-term for obesity management. It is a reversible inhibitor of gastric and pancreatic lipases, and causes reduced absorption of dietary fat (approximately 30% reduction). Its dosing does not involve titration and is currently available in 60 mg over-the-counter dose and 120 mg prescription doses. The dose should be taken three times daily with meals containing fat. Due to its mechanism of action, its side effects include oily spotting, flatus with discharge, fecal urgency, and bloating. It can also result in fat-soluble vitamin deficiencies, involving vitamins A, D, E, and K; therefore, daily supplementation is recommended two hours before or after orlistat administration. While it is a relatively effective drug (average two to three kilogram weight loss), it has a high discontinuation rate in studies (30–40%) due to its unfavorable side effect profile [42]. If weight loss is not seen after 12 weeks of use, discontinuation should be considered. It is contraindicated in those with chronic malabsorption syndrome or cholestasis. Drug-drug interactions with medications such as warfarin, cyclosporine, and levothyroxine need to be assessed prior to initiation. Due to low systemic absorption, no dose adjustments are needed in the setting of any hepatic or renal dysfunction. A small prospective double-blind randomized placebo-controlled trial in 2006 compared a group taking orlistat for 6 months and a placebo group. Both groups underwent a strict weight loss program and upon study completion, the authors concluded that the orlistat group had a greater percentage of patients with normal hepatic echo pattern on ultrasound compared to those of the control group; with a *p* of 0.04, the authors of this study concluded this observation was unlikely due to chance. However, histopathologic study revealed that these two groups showed similar improvement when analyzed under the microscope [43].

Liraglutide is a glucagon-like peptide-1 (GLP-1) analogue and an FDA-approved treatment for overweight or obese patients. It stimulates gastrointestinal peptides that stimulate glucose-dependent insulin secretion and inhibits glucagon release and gastric emptying. It is a once daily subcutaneous injection that involves titration to ensure tolerability of side effects. Side effects include nausea and vomiting; however, these side effects typically subside with time and can be avoided with proper counseling (eating smaller portions, eating more slowly, etc.). It is contraindicated in patients with a personal or family history of medullary thyroid cancer or endocrine neoplasia type 2. Its use is cautioned in patients with gastroparesis due to its delayed gastric emptying effects. Due to epidemiologic data suggesting possible risk of pancreatitis, use is also cautioned in patients with history of pancreatitis or severely elevated triglycerides. In patients with severe renal or hepatic dysfunction, dose adjustment is not necessary, however it should be used cautiously in these patients as there is limited data. Liraglutide has been studied in both diabetic and non-diabetic patients: A 20 week randomized trial of 464 patients comparing liraglutide, placebo, and orlistat showed that when comparing any dose of liraglutide with placebo, patients lost significantly more weight (mean loss of 2.8 kg) and when comparing high doses of liraglutide (2.4 and 3.0 mg daily) compared to orlistat, patients lost significantly more weight [44]. A retrospective study in diabetic patients showed that liraglutide use for 26 weeks was associated with decrease in weight and HbA1c levels, which mediated a decrease in ALT among subjects with elevated baseline ALT [45]. A recently published randomized double-blind placebo-controlled trial (2016) compared the use of liraglutide compared to placebo in NASH patients [46]. The authors found histologic resolution of NASH without worsening of fibrosis, concluding that the observation was unlikely due to chance alone. Liraglutide showed improvement in hepatic insulin sensitivity, reduction in hepatic endogenous glucose production, and improvement in adipose tissue insulin sensitivity. Significant weight loss was also noted in the liraglutide group compared to placebo. While these conclusions may be applicable to the general NAFL population, similar studies are lacking.

The choice for which obesity drug therapy to initiate should firstly be guided by avoiding contraindications and drug-drug interactions. Secondly, a therapy with a mechanism of action that targets the patient’s dietary behaviors should be chosen. For example, for patients that express difficulty with dietary cravings, combination buproprion-naltrexone is an effective option. For patients with problems with difficulty achieving satiety, lorcaserin would target satiety through its mechanism of action. For excessive appetite, phentermine-topiramate combination therapy is an option, and for pre-diabetic, liraglutide would provide glucose control in addition to weight loss. 

Lastly, regardless of the drug therapy chosen, close monitoring is important to assess efficacy and tolerability as well as promptly determine if therapy should be continued or switched to an alternative option. Since obesity is considered a chronic condition, if the drug therapy is effective, the medication should generally be continued chronically unless the patient cannot tolerate or has pregnancy plans [37]. The exception is with phentermine, which is only approved for short-term use (up to 12 weeks). 

## 7. Conclusions

Presumably, there are significant clinical and long-term benefits of preventing the development of NAFL into NASH and fibrosis. From a preventative medicine perspective, there remain millions of patients with NAFL who may convert into NASH which carries a higher risk of adverse outcomes including cirrhosis, end-stage liver disease, hepatocellular carcinoma and death, if liver transplantation is not pursued in a timely fashion. From an economic perspective, preventative methods often become more cost-effective overtime compared to managing sequelae of end-organ disease or treating advanced disease. Patients who have trialed lifestyle changes and have failed to lose ≥5% of their body weight have not exhausted all their options; for such patients with NAFLD, healthcare providers should consider a trial of FDA-approved anti-obesity medications as adjunct therapy to provide further preventative and therapeutic options as an effort to reduce the risk of NAFLD-related disease progression.

## Figures and Tables

**Table 1:** FDA approved anti-obesity medications.

Year	FDA-Approved Medications
1959	Phentermine
1959	Diethylpropion
1973	Benzphetamine
1976	Phendimetrizine
1999	Orlistat
2012	Phentermine + Topiramate ER (Qsymia)
2012	Lorcaserin (Belviq)
2014	Bupropion + Naltrexone (Contrave)
2014	Liraglutide (Saxenda)

**Table 2:** Anti-obesity medications withdrawn from the market.

Year	Medications and Reasons for Withdrawal
1972	Aminorex 1972: fatal pHTN
1979	Amphetamine 1979: addictive behavior and neurological SE
1997	Phen-fen (Phentermine + fenfluramine) 1997: mitral and aortic valvular heart disease and pHTN due to fenfluramine
1997	Defenfluramine 1997: see above
2004	Ephedra (FDA Ban) 2004: Fatal arrhythmias and HTN
2009	Rimonabant (Europe) 2009: Psychiatric illness and suicide
2010	Sibutramine 2010: Increased CV events and strokes
